# Research on the Design and Automatic Recognition Algorithm of Subsidence Marks for Close-Range Photogrammetry

**DOI:** 10.3390/s20020544

**Published:** 2020-01-19

**Authors:** Liyuan Meng, Jingui Zou, Guojian Liu

**Affiliations:** 1 School of Geodesy and Geomatics, Wuhan University, Wuhan 430079, China; 2 Key Laboratory of Precise Engineering and Industry, National Administration of Surveying, Mapping and Geo-information, Wuhan University, Wuhan 430079, China

**Keywords:** photogrammetry, barcode, image processing, tilting compensation, settlement survey

## Abstract

In China, traditional techniques for measuring structural subsidence cannot keep pace with the rapid development of critical national infrastructure such as the growing network of high-speed railways. Traditional monitoring methods using leveling instruments are inefficient and time consuming when monitoring structures like bridges and tunnels. Thus, a fast, economical, and more accurate and precise way to survey building subsidence is urgently needed to address this problem. This paper introduces a new close-range photogrammetry technique that deploys a fixed camera with tilt compensator to measure changes in height over small areas. A barcode subsidence mark that can be identified automatically during digital image processing replaces the leveling points used in traditional methods. Four experiments at different locations verified that results from the new method were stable and consistent with total station measurements. This approach is simple, inexpensive, and produces accurate and precise results as our evaluation results show.

## 1. Introduction

A growing number of public facilities such as the high-speed railways, subways, and underground corridors are under construction in China given the increased state investment in infrastructure construction. Given this rapidly changing and complex built environment, building settlement must be monitored in real time to ensure the safety and durability of structures during construction and operations. The commonly used methods are leveling, hydrostatic measurement, and sensors such as stress detectors [1]. Leveling is widely used; for example, the second-level leveling measurement technology is used to obtain the settlement of a mining area and reliable monitoring results are obtained and can reflect the actual settlement deformation law of the mining area [2]. Leveling points are set up in corridors of different elevations and the basic deformation characteristics of the dam foundation are acquired through long-term monitoring data [3]. Leveling is used to monitor foundation pit settlement and the settlements of the two most representative monitoring points are predicted [4]. But when in use, facilities like high-speed rail lines are difficult to approach, making leveling using manual measurements unsuitable in many instances. Furthermore, continuous manual infrastructure monitoring is time consuming and cannot meet the demanding requirements imposed by the pace of development [5]. Hydrostatic leveling overcomes the limitations of manual leveling and the instruments are buried in a structure to measure changes in height, A novel monitoring system using innovative fiber-optic liquid-level transducers is proposed for continuously monitoring the settlement of geotechnical infrastructures and its relative measurement error is within 4% [6]. The performance of a hydrostatic leveling system for monitoring the deformation of a power transmission tower base together with a slope sensor and displacement meter is evaluated. The monitoring results show that hydrostatic leveling measurements are strongly affected by the environmental temperature. Therefore, to obtain the actual deformation of a monitoring target, the measurements should be further processed to reduce the effect of temperature on the result [7]. What is more, the instruments of hydrostatic leveling contain liquid and can leak. Even if the liquid does not leak, the viscous liquid cannot measure fast-changing height variations rapidly. Methods of using sensors, such as a settlement plate and displacement sensor, all have drawbacks on instantaneity, sensitivity and accuracy and the results of those methods cannot be processed using the adjustment theory [8,9]. Therefore, a monitoring method for infrastructure with high accuracy and high efficiency is urgently needed to monitor infrastructure and ensure it is safe.

There are many advantages to close-range photogrammetry [10,11,12]. The first is that a large number of geometry structure information of the objects to be measured can be acquired instantly. A lot of visual information is recorded on the images, from which the measurement results can be derived. Photogrammetry increases the measurement accuracy and efficiency greatly when there are many measuring points on the object to be measured. The second advantage is that photogrammetry will not interfere in the state of motion or surface material of the object to be measured because it is a kind of non-contact measurement and photographing is non-destructive. The third advantage is that the camera can work in harsh environments that people cannot work in, such as underwater, near hot material and oxygen-poor zones. The forth is that photogrammetry can record the changes of the state of motion because of the instantaneity of the camera exposure. At any time images of the object measured can be taken and data of interest, such as space data, image data, dynamic sequence of images and even three-dimensional models, can be acquired after processing by the photogrammetry system.

The development of science and technology and the emergence of rigorous data-processing methods lay a stable theoretical and technical foundation for close-range photogrammetry. With the maturity of hardware and software for close-range photogrammetry and the further maturity of artificial intelligence, machine learning and other technologies, it is possible to monitor major national infrastructure using close-range photogrammetry. Close-range photogrammetry, as a measuring method, has been widely used in the fields of space information science, such as three-dimensional modeling, cultural relic protection, water resources protection and mining monitoring [13,14,15,16,17,18]. The plant volume is estimated by acquiring data for constructing a 3D model of a living plant using close-range photogrammetry [13]. Close-range photogrammetry is used to measure soil disturbances and it can give information not available via traditional methods [14]. A non-contact 3D optical static deformation measurement system, based on photogrammetric detection is applied to experimentally evaluate the structural properties of a large-capacity composite tank, allowing an accurate reconstruction of the 3D shape of the tank from a number of freehand digital pictures taken from different angles and positions [15]. An analysis approach is created to detect damage on three-dimensional models form digital photogrammetry, richer in information about depth and volume, which can identify and quantify damage on the surfaces [16]. Photogrammetric technology is applied to investigate the deformation characteristics of rock mass when it changes from open pit to underground mining. The monitored results of digital photogrammetry are compared with the dial indicators and the maximum error between these two was less than 0.02 mm [17]. A new approach for energetic analyses of traffic accidents against fixed road elements using close-range photogrammetry is produced and a real accident of a car against a fixed metallic pole is analyzed to obtain the collision speed and absorbed energy [18]. A device is developed that mainly contains a point light source and a point-position measurement device that uses a linear CCD (Charge-coupled Device) to detect the position change of the point light’s image. Any position change of the point light source can be measured and the settlement for one point can be obtained remotely [19]. However, the device cannot obtain the height difference between two points and the adjustment of the leveling line cannot be processed.

**Figure 1:**
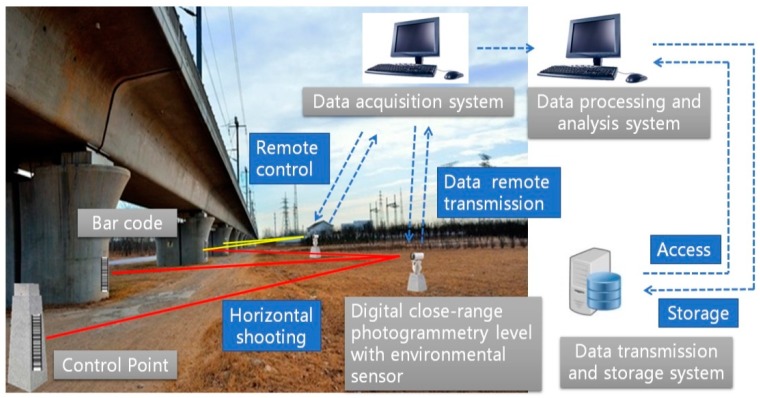
The architecture of the settlement monitoring system of photogrammetry.

A new architecture of the settlement monitoring system of photogrammetry is designed, as shown in Figure 1, taking the high-speed rail settlement monitoring as an example. The system is designed to consist of a data acquisition system, data-processing and analysis system, and data transmission and storage system [20,21]. Using the pan-and-tilt camera with environmental sensor of the data acquisition system, the images of the barcode installed on the pier can be taken and sent to the data-processing and analysis system. The digital close-range photogrammetry level with environmental sensor can decide whether to start monitoring based on the data of environmental sensor. The images are processed and recognized in the processing and analysis system and the heights of the monitoring points are acquired. The settlement value of the pier can be obtained by comparing data of multiple periods and the subsidence trend of the pier can also be analyzed. Using data transmission and a storage system can realize the data storage, search and report generation. Monitoring a railway bridge using close-range photogrammetry can not only save a lot of manpower and resources but also shorten the working period to ensure the safe operation of the high-speed railway. The height difference of two bar codes on the different piers can be measured. Bringing in the starting and ending control points, the adjustment of the leveling line can be processed. Situations of the area can be monitored in real time through the images and the results are easily visible. Using a high-speed camera can also monitor the bridge vibration in the future.

In order to achieve automatic and real-time monitoring, it is first necessary to realize real-time and automatic measurement of elevation. The close-range photogrammetry subsidence mark, which is critical to achieving real-time and automatic measurement, is designed in the article and the method to recognize and locate the mark is put forward and implemented by integrating it with several image-processing algorithms. The method of tilt correction is also proposed and implemented. All the methods proposed in the paper are verified by experiments. The research results of the article lay the foundation for a settlement-monitoring system of close-range photogrammetry and provide a new solution to engineering monitoring.

## 2. Materials and Methods

### 2.1. Subsidence Mark Design

**Figure 2:**
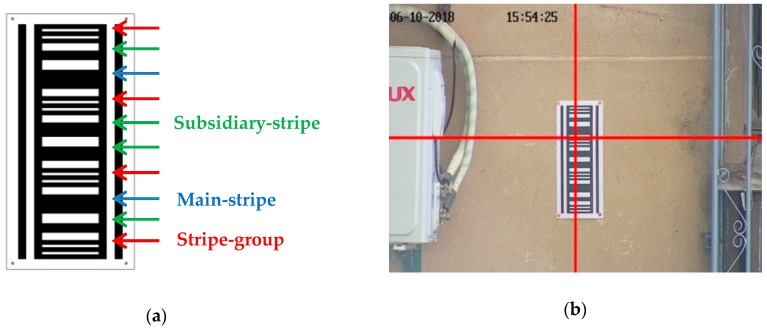
Subsidence mark, (**a**) subsidence mark which is designed as a barcode; (**b**) the image shoot during the monitoring.

In order to obtain the subsidence automatically, it is necessary to design the measuring mark that both provides necessary mathematical information and is easily recognized. The measuring mark must be readable so that the results can be verified manually [22]. The common settlement dot marks are designed for a levelling rod, which cannot be used for photogrammetry. For photogrammetry there are ordinary round markers and coding markers. Coding markers designed by the other researchers are used for finding the mark quickly and matching the image efficiently, while the mark we need is specifically designed for the settlement monitoring using photogrammetry. A barcode is designed as the subsidence mark, as shown in Figure 2a. The mark consists of black and white horizontal stripes. There are 12 black pin stripes, which form four groups of three from bottom to top, which are called stripe-groups here. The height of the stripe-group equals the height of the stripe in the middle in every stripe-group. The vertical width of the stripe-group equals the vertical width from the bottom of the bottom stripe to the top of the top stripe in the group. According to the design data, the heights of the stripe-groups are 0 mm, 90 mm, 180 mm and 270 mm, respectively. There are 2 black stripes between every two stripe-groups and 6 in total, the boldest two are called main-stripes and the others are called subsidiary-stripes. The vertical-width of the main-stripe equals the vertical-width of the group-stripe. The arrangement of the main-stripe and subsidiary-stripe can help identify the barcode. The barcode mark is like a ruler, and contains math information, so that the height of each position of the barcode can be read. Another superiority of the mark is it is easy to recognize and is not easily confused with other objects.

The camera is installed on the observation pillar and the photographic baseline is level under ideal conditions, then the point on the mark corresponding to the photograph center and the photograph center are on the same level, as shown in Figure 2b. The red cross shows the center of the image and it must be in the region of the mark by adjusting the initial position of the camera, which can help in detecting the region of interest (ROI). The height of the photograph center can be calculated from the barcode as designed. When the structure monitored is stable, the height reading of the photograph center from the barcode is stable, too. The difference between the height readings at different times can reflect the settlement changes of the structure accurately.

Not only can the settlement changes be identified but the height difference between the two neighboring barcodes monitored by the same camera can be calculated as well since the camera is installed on a platform which can rotate in the horizontal plane. There are two control points set up near the first monitoring point and the last monitoring point so that the heights identified from the photograph can be corrected through the adjustment of the leveling network.

### 2.2. Subsidence Mark Recognition

The purpose of the subsidence mark recognition is to find the barcode on the image and determine the height of the photograph center through the height reading from the barcode. Many algorithms, such as Sobel edge detection, Canny edge detection and Hough transformation are used during recognition.

**Figure 3:**
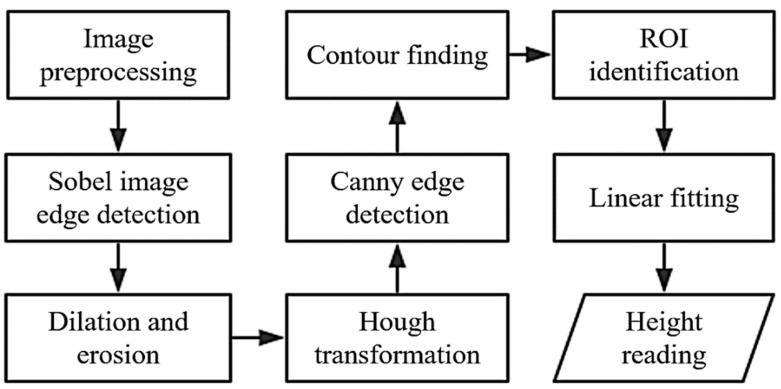
The image processing flow, preprocessing image preprocessing includes gray processing and Gaussian smoothing.

The image-processing flow is shown in Figure 3. The first step is the image preprocessing which includes gray processing and Gaussian smoothing. Then transverse Sobel operator is used for transverse edge detection, and dilation and erosion are used to find the region of the mark, which includes the image center. The region of the mark is the ROI. Then the ROI is widened and the lines in the ROI detected using Canny edge detection and Hough transformation. The lines belonging to the barcode remain by deleting the lines with a big slope or far from the other lines. Finally the main-stripes, the subsidiary-stripes and the stripe-groups are distinguished according to the vertical width ratio and arrangement, so that the height of the photograph center can be calculated using linear fitting. Although some of the algorithms used during the image processing are classic algorithms, the novelty of the algorithm lies in the recombination of multiple algorithms to achieve the purpose of identifying the designed barcodes autonomously.

#### 2.2.1. Sobel Edge Detection

Sobel edge detection is the edge detection algorithm using Sobel operator, the finite-difference operator, which can produce a luminance difference approximation for edge detection [23]. The operator can be divided into two types, lateral detection operator and longitudinal detection operator, either of which can be expressed by a matrix of three rows and three columns. R is used to express original image, and $G_{x}$ and $G_{y}$ are used to express images after edge detection, their relation is shown in Formulas (1) and (2).

(1) $$G_{x}=\left[\begin{array}{cc} \begin{array}{c} -1\\ -2 \end{array} & \begin{array}{cc} 0\; & 1\;\\ 0\; & 2\; \end{array}\\ -1 & \begin{array}{cc} 0\; & 1\; \end{array} \end{array}\right]*R$$

(2) $$G_{y}=\left[\begin{array}{cc} \begin{array}{c} 1\\ 0 \end{array} & \begin{array}{cc} 2 & 1\\ 0 & 0 \end{array}\\ -1 & \begin{array}{cc} -2 & -1 \end{array} \end{array}\right]*R$$

Because the stripes on the barcode are horizontal stripes, Sobel lateral detection operator is used so that a lot of information unwanted can be filtered out. As shown in Formula (3), f(x,y) expresses the pixel value at position (x,y).

(3) $$\begin{array}{l} G_{x}=\left(-1\right)*f\left(x-1,y-1\right)+0*f\left(x,y-1\right)+1*f\left(x+1,y-1\right)+\\ \left(-2\right)*f\left(x-1,y\right)+0*f\left(x,y\right)+2*f\left(x+1,y\right)+\left(-1\right)*f\left(x-1,y+1\right)\\ +0*f\left(x,y+1\right)+1*f\left(x+1,y+1\right)\\ \;=\left[f\left(x+1,y-1\right)+2*f\left(x+1,y\right)+f\left(x+1,y+1\right)\right]\\ \;-\left[f\left(x-1,y-1\right)+2*f\left(x-1,y\right)+f\left(x-1,y+1\right)\right]. \end{array}$$

**Figure 4:**
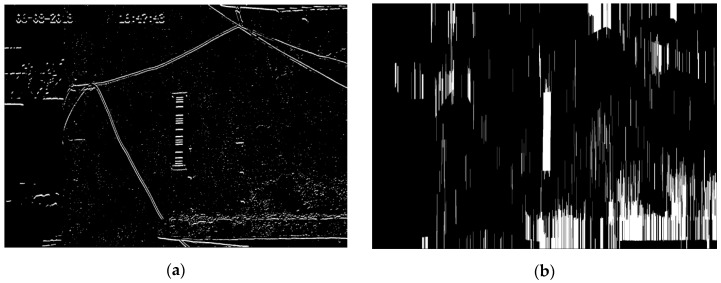
Mark location confirmed, (**a**) Sobel transverse edge detection; (**b**) the image after the operation of erosion, dilation and erosion. The white region in the middle of the image is the region of the stripes.

The image after Sobel lateral edge detection is shown in Figure 4a and the information of the horizontal stripes remains completely. The operation of erosion and dilation is used many times. The first operation of erosion can remove small fragments and vertical dilation can interconnect the stripes of the barcode because of the vertical alignment of the stripes. The second operation of erosion is used to make the region of the stripes return to its original size. The image after the operation of erosion and dilation is shown in Figure 4b, in which the white region in the middle of the image is the region of the barcode.

#### 2.2.2. Canny Edge Detection

**Figure 5:**
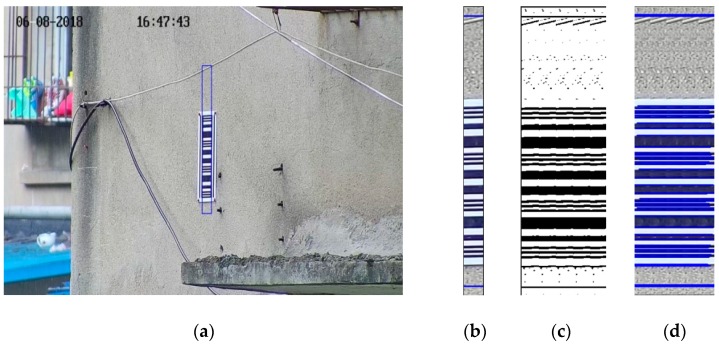
The process of the line detection on the mark, (**a**) the region of the mark recognized on the image; (**b**) the remaining middle part of the region of interest (ROI); (**c**) five RIOs; (**d**) the line extraction result of Hough transformation.

The region of the barcode can probably be determined after Sobel edge detection, as shown in Figure 5a, in which the region in the box is the ROI, which will be processed next. The left quarter and red quarter of the ROI is abandoned since it may influence the result of Canny edge detection because of the distortion and mismatching. The remaining middle part of the ROI is too narrow to be detected, as shown in Figure 5b. Five RIOs are put together and then the width of the ROI is enough for detection, as shown in Figure 5c. Another advantage of splicing is that it can reduce orthographic distortion.

Canny edge detection is used in Figure 5c. Canny edge detection is a multi-level detection algorithm whose basic idea is to find the position where the gray level change is the strongest in the image. The derivatives of the horizontal and vertical directions are calculated using Canny operator and the gradient and direction of the edge can be calculated, as shown in Formulas (4) and (5). When the gradient and direction are calculated, every pixel on the image is traversed and the point with the biggest gradient among the points with the same direction is reserved, which is the boundary point [24].

(4) $$E\_G=\sqrt{G_{x}^{2}+G_{y}^{2}}$$

(5) $$A=\tan^{-1}\left(\frac{G_{x}}{G_{y}}\right)$$

#### 2.2.3. Hough Transformation

Line detection from binary images is the basic Hough transform [25]. By transforming the image coordinate space into the parameter space, line fitting can be realized.

The line-fitting result of Hough transformation is shown in Figure 5d. The first and last stripes in the image are deleted because they are far from the other stripes. The stripes not belong to the barcode will also be deleted, and all the correct stripes remain.

#### 2.2.4. Height Calculating Using Linear Least Squares Fitting

The least squares method is a mathematical optimization method that finds the best function match of the data by minimizing the sum of the squares of the errors [26]. The least squares method can be used to easily obtain unknown data and minimize the sum of the squares of the errors between the obtained data and the actual data. The height of the middle pixel of the image is determined by linear least squares fitting of the height and the pixel v coordinates of stripe-groups. The results will be shown in Section 3.1.

### 2.3. Tilting Compensation

#### 2.3.1. The Tilt Correction Using an Electronic Tilt Compensator

**Figure 6:**
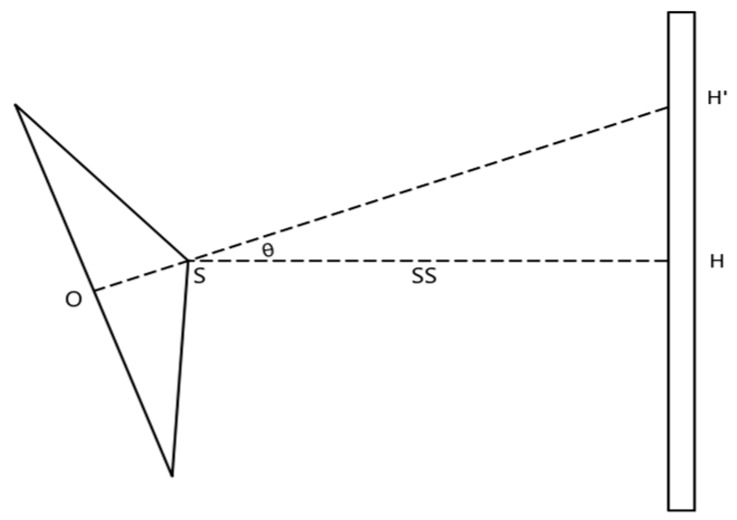
The influencing value from angle θ. O is the image center, S is the projection center, as OS is small enough, H and $H^{\prime }$. are the real value and measuring value of the height of the photograph center, respectively.

In actual measurements, photographic baseline will not be level absolutely. There is an angle θ between the photographic baseline and the horizontal direction, as shown in Figure 6. Angle θ will influence the result of the measurement, which is shown in Formula (6); HH’ is the influencing value on the height of the middle pixel of the image from angle θ; SS is the horizontal distance from the barcode to the projection center. Angle θ must be confirmed to correct the result of the measurement. It can be confirmed by an electronic tilt compensator, which is installed in the camera. The angle between the photographic baseline and the tilt compensator is also necessary and it can be calibrated [27]. The tilt compensator used is a dual-axis tilt sensor with ultra-precise and digital output function produced by Bewis Sensing. The parameters of the tilt compensator used are shown in Table 1.

(6) $${\mathrm{HH}}^{\prime }=\mathrm{SS}\cdot \tan\theta$$

#### 2.3.2. The Tilt Correction Taking Account of Error from the Barcode

**Figure 7:**
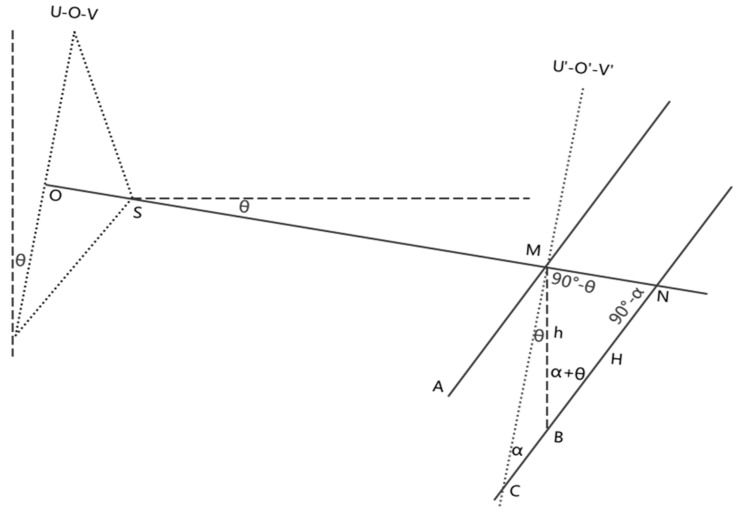
The central projection when the photographic baseline is non-level and the barcode is not vertical. The geometrical relationship of relevant parameters before and after settlement is also shown.

When the measuring mark is installed, the plane of mark cannot be strictly vertical, which will influence the result of the settlement. As shown in Figure 7, Plane U-O-V is the image plane and O is the principal point on Plane U-O-V, S is the projection center and the angle between the photographic base and the horizontal direction is θ. Plane U′-O′-V′ is the plane which is parallel to the Plane U-O-V and contains Point M. AM shows the starting position of the measuring mark and it is not vertical; there is an angle α between AM and Plane U′-O′-V′, then the angle between AM and the vertical direction equals to θ+α. CN shows the position of the measuring mark after settlement. As shown in Figure 7, Point M moves to Point B after settlement, the actual settlement value is h. However, the settlement value measured by the image is H since the readings from the barcode on the image are M(B) and N respectively.

As shown in Figure 7, a triangle is formed by lines joining points BMH. Based on the geometric relation and the sine law, the following equation is satisfied:

(7) $$\frac{h}{\sin\left(90{}^{\circ}-\alpha \right)}=\frac{H}{\sin\left(90{}^{\circ}-\theta \right)}\;$$

The rearranged equation is as follows:

(8) $$h=H\cdot \frac{\cos\alpha }{\cos\theta }$$

**Figure 8:**
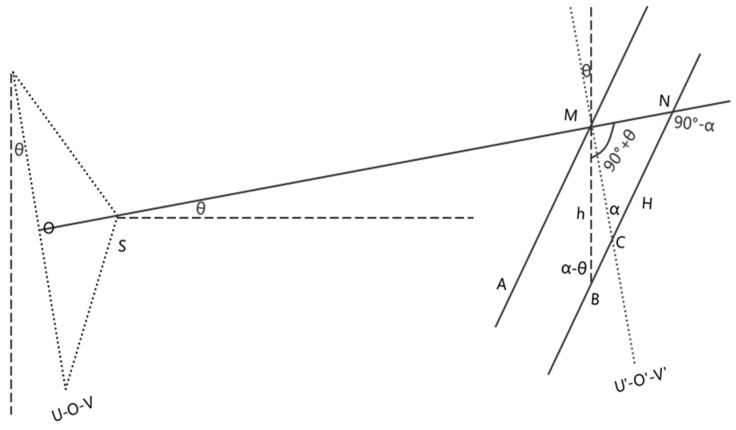
The central projection when the photographic base is non-level and the bar code is not vertical in another condition. The geometrical relationship of relevant parameters before and after settlement is also shown.

Since angle θ. can be the angle of depression or elevation and angle α angle also can be the angle with two different directions, and either angle can be larger than the other, there are several other conditions of the geometric relations. As shown in Figure 8, it is another condition that angle θ is angle of elevation and angle α is larger than angle θ. In Figure 8, a triangle is also formed by lines joining points BMH and based on the geometric relation and the sine law, the following equation is satisfied:

(9) $$\frac{h}{\sin\left(90{}^{\circ}-\alpha \right)}=\frac{H}{\sin\left(90{}^{\circ}+\theta \right)}$$

The rearranged equation is the same as Equation (8). It can be deduced that in other conditions, the relationship of the actual settlement value h and the measuring settlement value H is also the same as Equation (8). Thus it can be concluded that when the angle between the photographic baseline and the horizontal direction is θ and the angle between measuring mark and image plane is α, the actual settlement value h can be calculated by correcting the measuring settlement value H using Equation (8).

In Equation (8), h is the unknown quantity to be evaluated and H is determined by the different readings before and after settlement, angle θ is measured using the electronic tilt compensator introduced in Section 2.3.1. Angle α is determined by the elements of exterior orientation of the camera based on the central projection theory of photogrammetry. The method and theory to determine α is described below.

According to the pinhole camera model and the fundamental projective geometry, the relationship of point Q in the physical world and its projection, point q, on the image is shown in Equation (10):

(10) $$\left[\begin{array}{c} x\\ y\\ 1 \end{array}\right]=sM\left(\begin{array}{cc} \begin{array}{cc} r_{1} & r_{2} \end{array} & \begin{array}{cc} r_{3} & t \end{array} \end{array}\right)\left[\begin{array}{c} \begin{array}{c} X\\ Y \end{array}\\ \begin{array}{c} Z\\ 1 \end{array} \end{array}\right]\;$$

where ${\left[\begin{array}{ccc} x & y & 1 \end{array}\right]}^{T}$ is the homogeneous coordinates of point q on the image plane, which is measured from the image. ${\left[\begin{array}{cc} \begin{array}{cc} X & Y \end{array} & \begin{array}{cc} Z & 1 \end{array} \end{array}\right]}^{T}$ is the homogeneous coordinates of point Q in the physical world, which can be given after the establishment of the physical world coordinate system based on the design of the barcode. s is an arbitrary scale factor. M is the matrix of the camera intrinsic parameters as shown in Formula (11):

(11) $$M=\left[\begin{array}{ccc} f_{x} & 0 & c_{x}\\ 0 & f_{y} & c_{y}\\ 0 & 0 & 1 \end{array}\right].\;$$

where $f_{x}$ and $f_{y}$. are the focal distances in pixels. The physical focal distance of the camera in millimeters is fixed, while the shape of each picture element is not a square but a rectangle, so there are two focal distances; one is in x direction and the other is in y direction. Parameters $c_{x}$ and $c_{y}$ are the coordinates of the principal point, which can indicate the deviation from the camera principal optic axis to the center of the imager [28].

**Figure 9:**
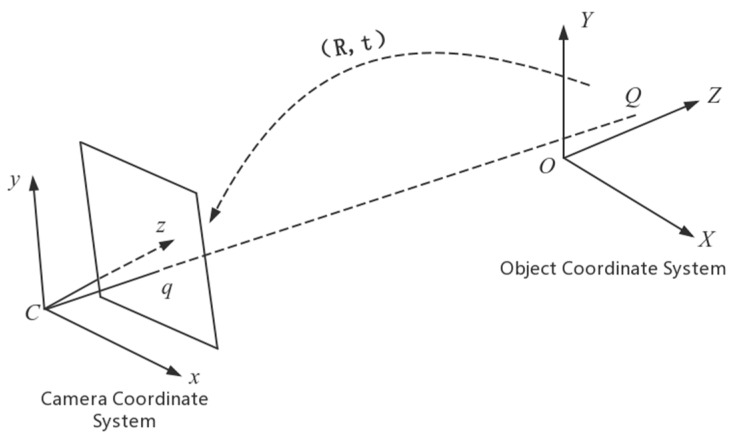
The translation and rotation relationship of the object coordinate system and the camera coordinate system.

In Equation (10), Parameter t is the translation vector, which indicates the deviation from the origin of the camera coordinate system including point q to the origin of the object coordinate system including point Q. $R=\left[\begin{array}{ccc} r_{1} & r_{2} & r_{3} \end{array}\right]$ is the rotation matrix, where $r_{1},\;r_{2},r_{3}$ are three vectors with three rows and one column. Using the rotation matrix R can make the object coordinate system revolve around the three axises successively and make the three axises of the object coordinate system parallel to the three axises of the camera coordinate system, respectively. As shown in Figure 9, Point q is on the image and the x axis and the y axis of the photo coordinate system parallel to the x axis and the y axis of the camera coordinate system, respectively. When the plane XOY of the object coordinate system is established using the plane of the barcode, the angle that the coordinate system revolves around the x axis over is the angle between the image plane and the barcode plane and that is the angle α exactly needed.

**Figure 10:**
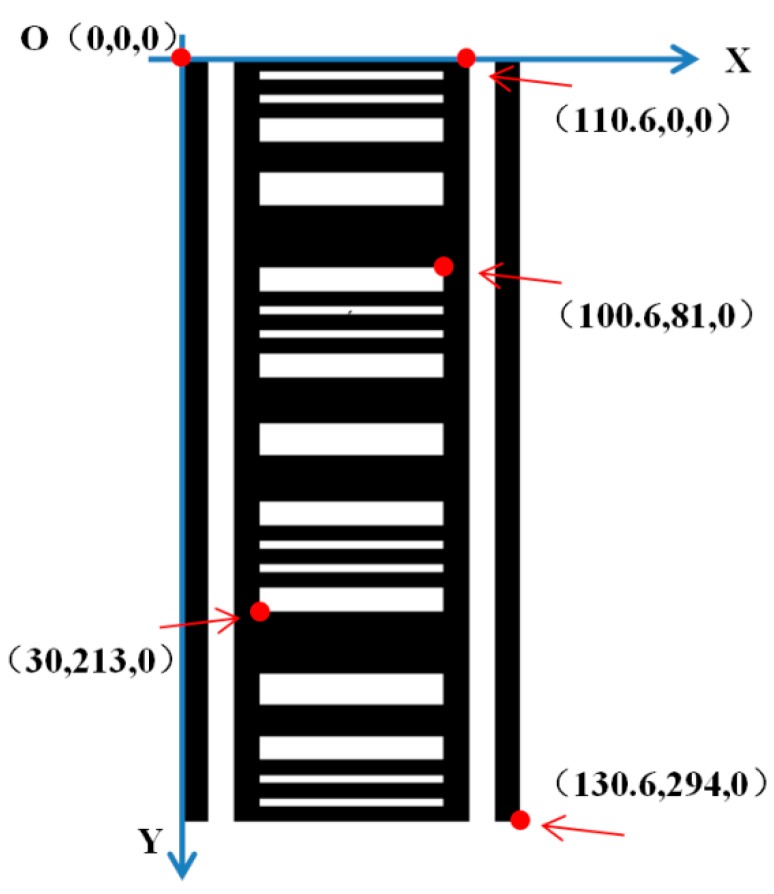
The object coordinate system established using the first point in the top left corner on the bar code as the origin. Some object coordinates of the points marked in red are shown as example.

As shown in Figure 10, the first point in the top left corner on the bar code is set as the origin of the object coordinate system, the coordinates of which are (0, 0, 0). The object coordinate system is established using the OX shown in Figure 10 as the x axis and the OY shown in Figure 10 as the y axis, then the direction perpendicular to the x axis and y axis is the z axis. According to the spatial relationships and the design data of the points on the bar code, the object coordinates of the 80 points on the barcode can be acquired. Since they are all in the plane XOY, the z coordinate of each point is 0. The object coordinates of some points is shown in Figure 10 as an example. The photo coordinates of the 80 points on the barcode can be collected from the image of the barcode. Since the x axis and the y axis of the photo coordinate system are parallel to the x axis and the y axis of the camera coordinate system respectively, the x coordinates and the y coordinates of the camera coordinate system and the photo coordinate system are the same. By plugging the coordinates of the 80 points in two coordinate systems into Equation (10), the unknown quantities, M, R and t, can be solved. Making use of the rotation matrix M, the angle that the coordinate system revolves around the x axis over can be calculated, which is exactly angle α. There are actually 11 unknown quantities in Equation (10), they are two focal distances, two coordinates of the principal point, three rotation angles, three translation parameters and one scale parameter, so at least four points are necessary to solve the equation. 

The rotation matrix is expressed as $R=\left[\begin{array}{ccc} r_{11} & r_{12} & r_{13}\\ r_{21} & r_{22} & r_{23}\\ r_{31} & r_{32} & r_{33} \end{array}\right]$, then the method of calculating the angles that the coordinate system revolves around the axises over from the rotation matrix is shown as Formula (12):

(12) $$\begin{array}{ll} \alpha = & \mathrm{atan}2\left(r_{32},r_{33}\right)\\ \beta = & \mathrm{atan}2\left(-r_{31},\sqrt{r_{31}{}^{2}+r_{33}{}^{2}}\right)\\ \gamma = & \mathrm{atan}2\left(r_{21},r_{11}\right) \end{array}$$

where α, β and γ are the angles that the coordinate system revolves around the x axis, y axis and z axis over respectively, atan2(a,b) is the function of C++, the result of which is atan(a/b) when the absolute value of b is larger than the absolute value of a and atan(b/a) when the absolute value of a is larger so that the function value is stable.

### 2.4. Horizontal Distance Calculation

The horizontal distance from the barcode to the projection center is necessary for height correction as shown in Formula (6). Referring to the measurement of the rotation angles from the rotation matrix, the horizontal distance from the barcode to the projection center can be calculated using the translation vector t, which indicates the deviation from the origin of the camera coordinate system to the origin of the object coordinate system. Translation vector t is a column vector with three elements, which can be expressed as $t={\left[\begin{array}{ccc} t_{1} & t_{2} & t_{3} \end{array}\right]}^{T}$ and the distance from the origin of the camera coordinate system to the origin of the object coordinate system is equal to the norm of t, which is expressed as $\left|t\right|=\sqrt{t_{1}{}^{2}+t_{2}{}^{2}+t_{3}{}^{2}}$.

**Figure 11:**
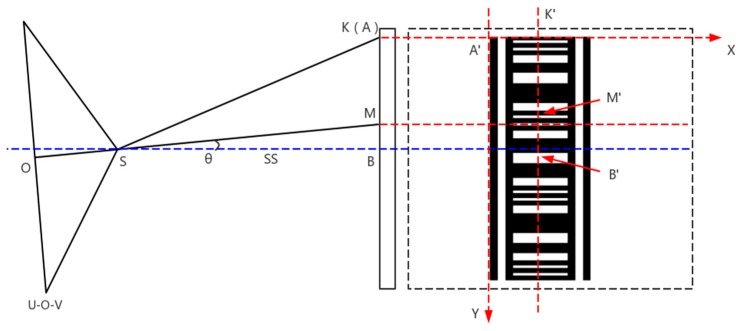
The relationship of the horizontal distance from the bar code to the projection center and the relevant geometric lines and points in the camera and object coordinate systems.

As shown in Figure 11, plane U-O-V is the image plane and O is the principal point on plane U-O-V. Point S is not only the projection center but the origin of the camera coordinate system as well. Points A, M and B are the points on the barcode in the physical world. Point M is the point of intersection of the photographic baseline and barcode. Point B is the point of intersection of the horizontal line through the projection center S and barcode and the distance from point S to B is the horizontal distance from the projection center to the barcode which is expressed as SS. Point A is the first point in the top left corner on the barcode and the origin of the object coordinate system. Point K is the point of intersection of line BM and the X axis of the object coordinate system and from the side view point K overlaps point A. The bar code in the dotted box is the bar code from the front view and point A′, M′, B′ and K′ are the same point to point A, M, B and K respectively.

In Figure 11, point A is the origin of the object coordinate system and the vector from point A to point S is $t={\left[\begin{array}{ccc} t_{1} & t_{2} & t_{3} \end{array}\right]}^{T}$, then the object coordinates of point S are $\left(t_{1},\;t_{2},\;t_{3}\right)$. Point M is the center of the image and by plugging the photo coordinates of the image center into Formula (10) the object coordinates of point M can be calculated, which is expressed as $\left(m_{1},\;m_{2},\;m_{3}\right)$ and the distance of line SM is shown in Formula (13):

(13) $$\left|\mathrm{SM}\right|=\sqrt{{\left(t_{1}-m_{1}\right)}^{2}+{\left(t_{2}-m_{2}\right)}^{2}+{\left(t_{3}-m_{3}\right)}^{2}},$$

Then the horizontal distance from the bar code to the projection center is as follows:

(14) |SS|=|SM|cosθ. 

## 3. Results

### 3.1. The Result of Subsidence Mark Recognition

The image-processing procedure and the method of height reading have been introduced in Section 2.2. Taking an image of a barcode as an example, the pixel *v* coordinates of stripe-groups are 365.167, 461.083, 556.833 and 652.167 in pixels, respectively, and the height of the stripe-groups in the physical world are 0, 90, 180 and 270 in millimeters respectively. Using linear least squares fitting the relationship of the height and the pixel *v* coordinates is shown in Formula (15):

(15) H=−0.940683×v+613.631 

The coordinates of the image center is decided by the image size and in the example the image width is 1940 in pixels and the height is 1080 in pixels, then the v coordinate of the image center is 540 in pixels, plugging which into Formula (15) the height of point on the barcode corresponding to the image center is 105.7 in millimeters. Using that method, the heights of the barcode in a leveling line can be all calculated. There are five bar codes in the leveling line and the height of each barcode is measured 10 times and then the average of the 10 heights is calculated as the final result, as shown in the second column in Table 2.

### 3.2. The Result of Tilting Compensation

#### 3.2.1. The Tilting Compensation Result of the Electronic Tilt Compensator

**Figure 12:**
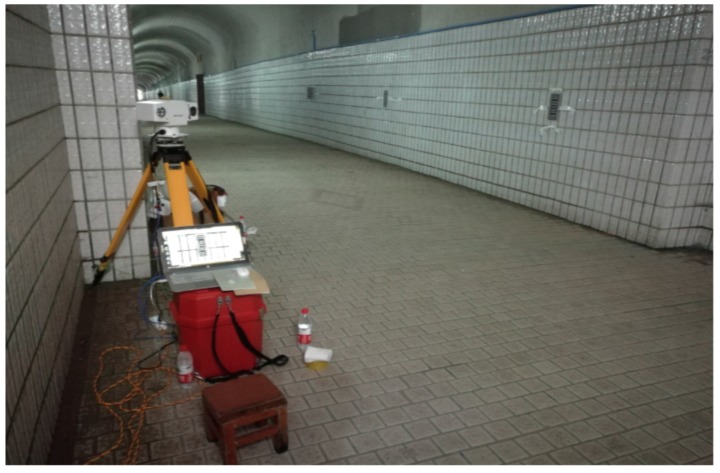
The leveling measurement field in a tunnel using close-range photogrammetry.

The method of compensating the height error from the angle between the photographic baseline and the horizontal direction using the electronic tilt compensator is shown in Formula (6). What calls for special attention is that the angle between the photographic baseline and the tilt compensator is 28′6.978″. The experimental data are acquired in a tunnel as shown in Figure 12. There are 5 monitoring points of the leveling line and the angles of the tilt compensator and the heights after tilting compensation are shown in the third and fourth columns in Table 2.

#### 3.2.2. The Tilting Compensation Result Taking Account of Error from the Barcode

As mentioned in Section 2.3.2, the tilt of the barcode influences the monitoring result of the height difference of one monitoring point. A barcode is hung on a slope with a small and fixed tilt and there is an initial position of the barcode named position 1 and another five positions are determined to form five height differences. Making use of the electronic tilt compensator and the angle between the measuring mark and the image plane, the height difference of the measuring point at different times can be corrected. As shown in Table 3, the second column is the height reading of each position, the third column is the non-corrected height difference calculated using the height readings in the second column, the fourth column is the pitch angle of the electronic tilt compensator, the fifth column is the angle between the measuring mark and the image plane which is named α, the sixth column is the corrected height difference, and the last column is the actual height difference measured by the total station. The height difference between position 1 and position 2 is shown in the same row as position 2 and so are the others. The pitch angles of the electronic tilt compensator are almost the same because the camera is fixed and static during the experiment. Angle α changes at neighboring positions and the average is used to correct the height difference.

### 3.3. The Result of the Horizontal Distance Calculation

According to the method in Section 2.4, the distance from the camera to the barcode is calculated using the elements of exterior orientation. The distances from the calculating result and the rangefinder are both shown in Table 4. The first row is captured at the position very close to the barcode and there is no optical zoom. The second row is captured at the distance of about 7 m from the barcode and there is a 3.0× zoom for the image identifiability of the barcode. The third row is captured at the distance of about 9 m from the barcode and there is a 25× zoom for the image identifiability of the barcode. As shown in Table 4 influenced by many factors, such as the focusing error, zooming error and the algorithm robustness, the distance from the calculating result cannot take the place of the distance from the rangefinder when there is a 25× zoom and some other experiments shows that the method of calculating the distance using the elements of exterior orientation cannot be applied to engineering at the moment.

## 4. Discussion

### 4.1. Measuring Stability Analysis

#### 4.1.1. Reading Stability on the Barcode When the Camera Lens Is Static

**Figure 13:**
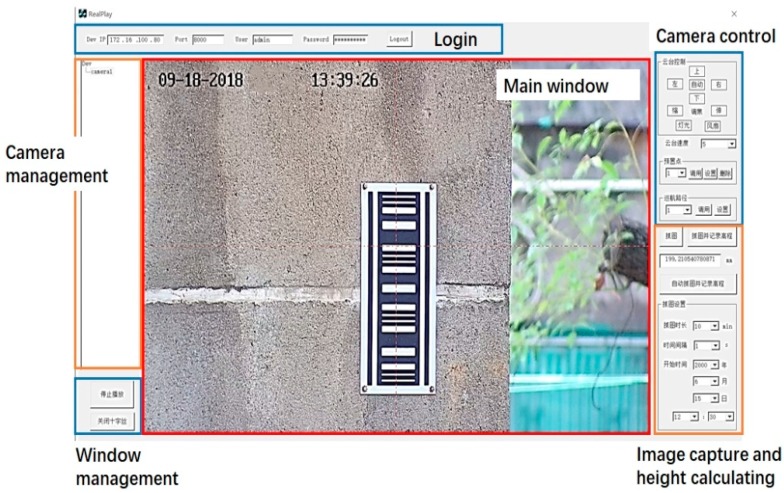
The interface of the software for the barcode recognition and height calculation.

The sensor used in the experiments was the PTZ camera of HIKVISION. The image sensor was 1/2.8” Progressive Scan CMOS, and the image size was 2048 × 1536. In order to realize the barcode recognition and height calculation, the SDK of HIKVISION who produced the camera used for measuring was loaded in MFC (Microsoft Foundation Classes) in the VS2017 development environment. The interface of the software achieving the aforementioned functions is shown in Figure 13, including the login module, main window, camera management module, window management module, camera control module, and image capture and height calculating module. After inputting the ID and password of the camera in the login module, the camera can be added to the camera management module and the images taken by the camera can be displayed in the main window in real time. The control of the camera such as the rotation and the zoom is realized in the camera control module and the camera parameters of every measuring point like the rotation angle can be set up in advance for automatic measurement in the future. In the image capture and height-calculating module, the height data can be automatically calculated and saved at regular intervals. 

**Figure 14:**
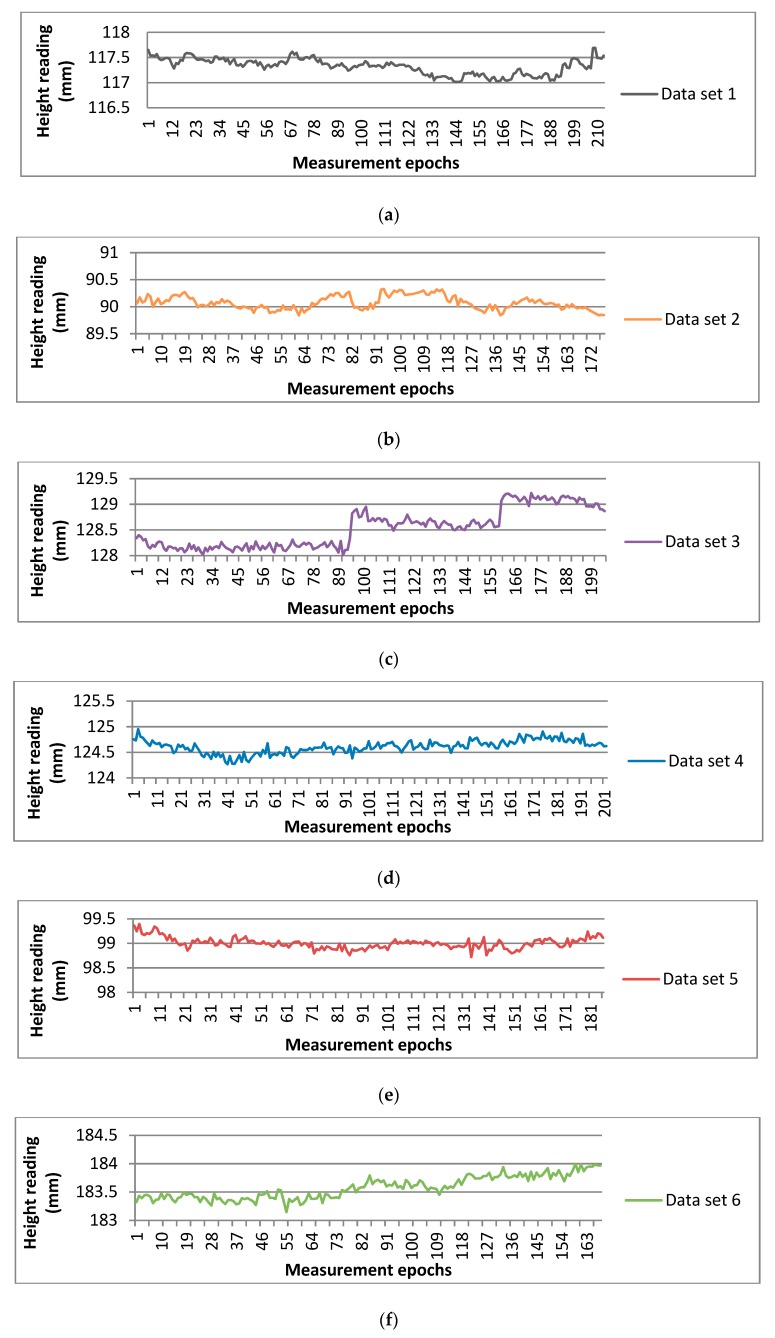
The data sets of the barcode height readings in different environments when the camera is static, (**a**) Data set 1; (**b**) Data set 2; (**c**) Data set 3; (**d**) Data set 4; (**e**) Data set 5; (**f**) Data set 6.

The barcode was measured in different environments to indicate the stability and reliability of the algorithm, including at different distances, from different angles and in different illumed conditions. The normal camera was used in a high light condition and infrared night vision was turned on in a low light condition like at night since the equipment used in the experiment has infrared night vision. Each set of data includes more than 200 readings and the standard deviations were calculated to evaluate the stability. During the measuring of one set of data, the parameters of the camera were fixed to eliminate the influence from the instability of the pan-and-tilt camera. There were six sets of data measured in different environments which are described using six different colors in Figure 14. The six environments are listed in Table 5; for example, the first was measured from the front side in a distance of 30 m and in high ambient light conditions and the sixth was measured from the right side with a small angle to the front in a distance of 50 m and in low ambient light conditions. As shown in Figure 14, the data sets in different environments were all taken on the feature of casual fluctuation around a certain value and the fluctuation range was less than 1 mm. The standard deviations of the six data sets is listed in Table 5 and it can be seen from the data that the conditions that the data sets measured from the front side were more stable than the data sets measured from side, and hence a conclusion can be reached that the stability of the barcode readings is mostly influenced by the viewing angle but less by the distance and light condition as long as the images are clear enough to be recognized.

#### 4.1.2. Reading Stability on the Barcode When the Camera Lens is Rotatable

In practical applications, the camera lens must be rotatable to measure the level difference of two monitoring points, which is necessary for the leveling as introduced in Section 1. The pan-and-tilt camera produced by HIKVISION can record the positions of several monitoring points and after one measurement period ends, the camera rotates back to the first monitoring point and a new measurement period begins. Therefore it is crucial that the pan-and-tilt camera is stable enough to make sure it can rotate back to the same positions of monitoring points as the previous periods.

**Figure 15:**
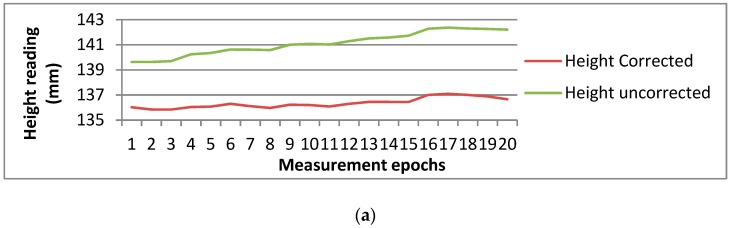

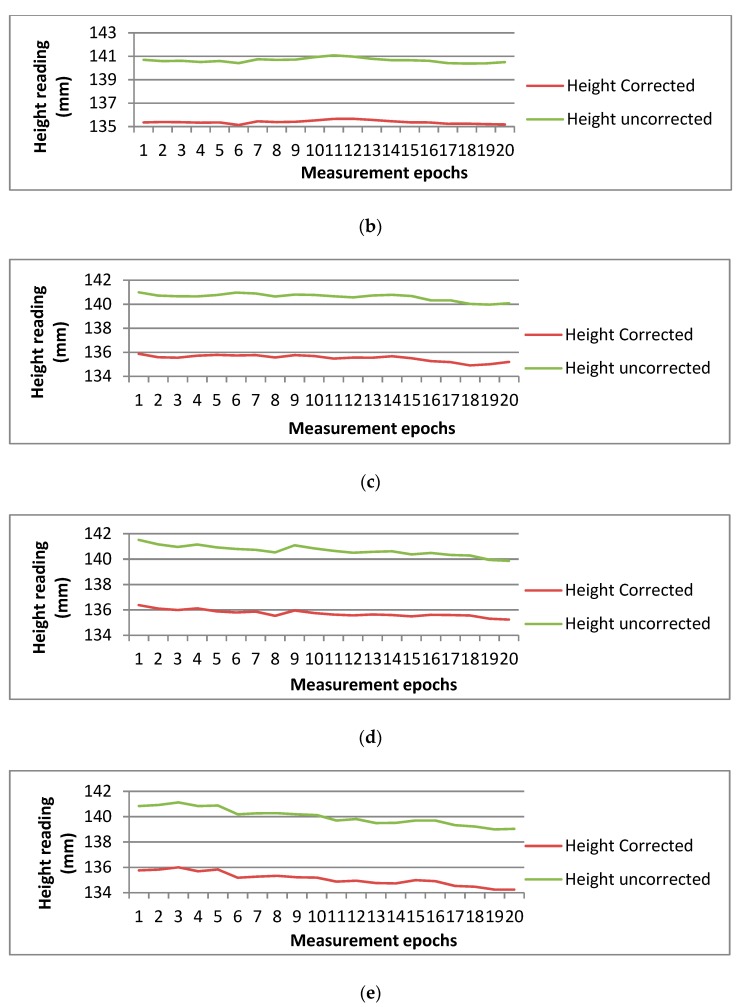
The variation trends of the 20 reading results of the five data sets, (**a**) Data set 1 which corresponds to 30 degrees; (**b**) Data set 2 which corresponds to 60 degrees; (**c**) Data set 3 which corresponds to 120 degrees; (**d**) Data set 4 which corresponds to 120 degrees; (**e**) Data set 5 which corresponds to 150 degrees.

The purpose of the experiment in this section was to verify the stability of the pan-and-tilt camera. The camera was installed at a distance of 30 m in front of the barcode and aims at the barcode first. The camera’s pose was recorded and then the camera was rotated at a certain angle and rotated back to the barcode using the records of the camera’s pose. After one minute’s standing, when the camera was stable, the measurement can be started and the stability of the pan-and-tilt camera can be verified from the measurement results. There were five data sets in the experiment. The first data set was acquired after the camera rotated 30 degrees and rotated back and the other four data sets corresponded to 60 degrees, 90 degrees, 120 degrees and 150 degrees respectively as shown in Table 6. During the acquisition of one data set the camera rotated away and back 20 times and when the camera rotated back, the barcode was read more than 30 times and the average value of the 30 readings was taken as the reading results to eliminate the error from the reading. The average value of the 20 reading results was taken as the height result of one rotation in Table 6. The variation trends of the 20 reading results of the five data sets are shown in Figure 15.

As shown in Figure 15, the data sets which correspond to 30 degrees and 150 degrees show a rising or declining trend obviously while the data sets which correspond to 60 degrees, 90 degrees and even 120 degrees are relatively stable, which is reflected in the standard deviations in Table 6 that the standard deviations of Data set 1 and Data set 5 are both bigger than the standard deviations of Data set 2, Data set 3 and Data set 4. 

In order to explore the compensation effect to the pan-and-tilt camera stability of the tilt compensator, the measurement results are compensated for using the tilting data of the compensator. As shown in Figure 15a, the rising trend of the compensated data becomes slow but the whole variant trend does not significantly alter and neither do the other data sets in Figure 15. The standard deviations of the corrected data sets are smaller than the standard deviations of the data sets uncorrected. The standard deviation corresponding to 30 degrees is improved significantly, which illustrates that the compensation to the pan-and-tilt camera stability of the tilt compensator is effective especially when the camera stability is poor.

### 4.2. The Effect of the Tilting Compensation Result of the Electronic Tilt Compensator in a Leveling Line

In Section 3.2, there were 5 monitoring points of the leveling line and the heights of the barcodes were compensated for using the tilt compensator. The heights before and after the tilting compensation are shown in Table 2. In this subsection the compensation effect will be discussed.

During the experiment in Section 3.2, all the height differences between the neighboring barcodes were measured using the total station as the actual values, as shown in Table 7. The height differences before and after tilting compensation were both calculated and compared with the measuring results of the total station and the discrepancies are also shown in Table 7. As shown in Table 7, the discrepancies are positive or negative, which is the feature of accidental errors. The biggest discrepancy before tilting compensation is 4.9 mm and the residual standard error is 3.8 mm, while the biggest discrepancy after tilting compensation is 2.1 mm and the residual standard error is 1.4 mm, which illustrates that the tilting compensation using the tilt compensator can improve the measurement accuracy effectively.

## 5. Conclusions

A barcode was designed for height monitoring using photogrammetry and a new method combining many algorithms, such as Sobel edge detection, Canny edge detection, Hough transformation and least squares method, was put forward for barcode recognition and height calculation, which was proven to be efficient and available by experiments. 

The pan-and-tilt camera produced by HIKVISION was chosen for the photogrammetry leveling measurement and the stability of the pan-and-tilt camera was tested by an experiment. 

A tilt compensator was installed in the camera. The relative angle between the compensator and the camera was calibrated. The tilting compensation method was put forward and the compensation results were compared for with the measuring results of the total station, which was proven to be effective. 

A method to calculate the horizontal distance from the barcode to the camera was also proposed while the experimental results are not all that effective, so the research will be continued in the future.

## Figures and Tables

**Table 1:** Parameters of the tilt compensator.

Item	Parameter
measurement axis	X-Y ^1^
measurement range (degree)	±5
measurement accuracy (degree)	0.001 ^2^
resolving power (degree)	0.0005 ^3^
Temperature effect on zero (degree/degree centigrade)	±0.0007 ^4^

^1^ X and Y are perpendicular; ^2^ It is applicable at room temperature; ^3^ It is applicable when perfectly still; ^4^ It is applicable at the temperature from minus 40 degrees centigrade to 85 degrees centigrade.

**Table 2:** The heights of the bar codes in the levelling line.

Levelling Point	Height (mm)	Angle of the Tilt Compensator (degrees)	Horizontal Distance (mm)	Height after Tilting Compensation (mm) ^1^
SZ01	119.7	0.4286	10,275.47	123.3
SZ02	141.2	0.3597	6797.358	147.6
SZ03	108.5	0.2137	4113.152	117.6
SZ04	146.6	0.2741	4691.9	154.5
SZ05	151.1	0.4257	13,012.5	156.0

^1^ It is calculated using Formula (6) and the angle between the photographic base and the tilt compensator is necessary.

**Table 3:** The height differences at different positions of one measuring point.

Position	Height Reading (mm)	Height Difference (mm)	Pitch Angle of the Electronic Tilt Compensator (degrees)	Angle α (degrees)	Corrected Height Difference (mm)	Actual Height Difference (mm)
1	108.7	-	-	-	-	-
2	115.0	6.2	−2.568905	−3.29871	6.2	6.2
3	125.6	10.7	−2.568605	−5.8378	10.6	10.5
4	135.1	9.5	−2.550805	−6.1726	9.4	9.4
5	152.3	17.1	−2.551005	−2.6675	17.1	17.1
6	162.2	9.9	−2.569805	−1.97165	9.9	9.9

**Table 4:** The distance calculation.

Position	Translation Vector (mm)	Object Coordinates of Point M (mm)	Distance Calculated (mm)	Distance from Rangefinder (mm)
1	${\left[-68.89\;-66.12\;2939.68\right]}^{T}$	(40.883, 146.136, 0)	2940.9	3000
2	${\left[-62.99\;-104.54\;6707.08\right]}^{T}$	(24.081, 129.408, 0)	6707.8	6750
3	${\left[-64.88\;-71.11\;5584.03\right]}^{T}$	(56.307, 142.3953, 0)	5589.43	9000

**Table 5:** Height reading stability analysis when the camera lens is static.

Data Set	Distance (m)	Light Condition	Side	Times of Reading	Average Reading (mm)	Standard Deviation (mm)
1	30	high	front	213	117.3	0.1
2	30	high	right	177	90.1	0.1
3	30	low	front	205	128.5	0.4
4	50	high	front	202	124.6	0.1
5	50	high	left	186	99.0	0.2
6	50	low	right	167	183.6	0.2

**Table 6:** Height reading stability analysis when the camera lens is rotatable.

Data Set	Rotation Angle (degrees)	Times of Reading	Average Reading (mm)	Standard Deviation (mm)	Average Reading Corrected (mm)	Standard Deviation Corrected (mm)
1	30	20	141.1	0.9	136.4	0.4
2	60	20	140.7	0.2	135.4	0.1
3	90	20	140.6	0.3	135.5	0.3
4	120	20	140.7	0.4	135.7	0.3
5	150	20	140.0	0.7	135.1	0.5

**Table 7:** Height differences before and after tilting compensation.

Order Number	Height Difference of Total Station (mm)	Height Difference of Barcode before Compensation (mm)	Height Difference of Barcode after Compensation (mm)	Discrepancy before Compensation (mm)	Discrepancy after Compensation (mm)
1	−26.4	−21.5	−24.3	4.9	2.1
2	28.6	32.7	30.0	4.1	1.4
3	−35.9	−38.1	−36.9	−2.2	−1.0
4	−1.3	−4.5	−1.5	−3.2	−0.1
